# The System of the Provision of Oral Healthcare in the Republic of Cyprus and the Effect of the Economic Crisis

**DOI:** 10.3290/j.ohpd.a43002

**Published:** 2020-07-04

**Authors:** Chrystala Charalambous, Mamas Theodorou, Kenneth Anthony Eaton

**Affiliations:** a PhD Student, Open University of Cyprus, Cyprus, Greece. Participated in the design of the study and the preparation of the questionnaires, searched the literature, extracted and analysed the data and wrote the paper.; b Professor, School of Economics and Management, Open University of Cyprus, Cyprus, Greece. Participated in the design of the study and the preparation of the questionnaires; reviewed the manuscript.; c Visiting Professor, University of Leeds, Leeds, UK; Honorary Professor, University of Kent, Kent, UK; Adviser to the Council of European Chief Dental Officers. Advised on the structure of the paper, reviewed and edited it.

**Keywords:** Cyprus, economic crisis, oral health

## Abstract

**Purpose:**

To investigate recent developments in the provision of oral healthcare in Cyprus and the population’s oral health, with special reference to the impact of the recent economic crisis. Research design: cross-sectional study.

**Materials and Methods:**

Data from oral health surveys in Cyprus over the last 30 years were reviewed and ana­lysed together with policy documents. Information regarding oral health behaviour, dental visits and the consequences of the economic crisis on the latter was also obtained with the help of self-completed questionnaires by patients and dentists.

**Results:**

Although the overall level of oral health in Cyprus can be considered satisfactory, there were statistically significant variations between districts and different socioeconomic and ethnic groups. Beyond these variations, it seemed that the three-year economic crisis (2013–2016) had negatively affected the behaviour of the population in seeking dental care, reducing the frequency of visits to the dentist, and avoiding costly dental work, especially among those from the lower socioeconomic strata. This change in citizens’ behaviour led to a statistically significant decrease in dentists’ income in the private sector.

**Conclusions:**

The economic crisis brought about new difficulties and challenges for both the public and the private sectors of oral healthcare, and into the implementation of a new National Health System.

During the last 20 years, there has been a steady improvement in oral health in Cyprus, as a result of both economic and social development and constant improvement in the availability of oral health services. The continuous training of dentists, the extensive use of modern technology, and the entry of highly specialised new dentists to the market, may also have contributed to this positive change.

The collapse of the Cyprus banking system and the bail-in of the deposits (up to 90% lost) from the country’s two largest banks have led to a deep economic recession. In November 2012, a Memorandum of Understanding (MoU) was agreed and sighed between the Troika and the Cyprus government, which had to take tough economic measures and carry out structural changes and reforms to the economy and public administration.^11^ The economic crisis has affected the economy, leading to deep recession and high unemployment for the whole population with changes in consumer behaviour and the everyday life of citizens and patients. Austerity measures were also imposed in the healthcare sector through cuts in the Ministry’s budget and civil servants’ salaries, changes in the criteria for free access to the public healthcare system and introduction of copayments for some outpatient visits, pharmaceuticals and diagnostic examinations, including oral healthcare. More specifically, from August 2013, a €3 charge per visit to the dentist was introduced, while the fee for partial or complete dentures was increased from €75 to €100.^34^

It should be explained that one of the obligations emanating from the MoU is the implementation of the new National Health System, according to the principles of the founding law of 2001, since the current system suffers from many weaknesses and problems such as (a) lack of universal coverage; (b) overcapacity and underutilisation of the infrastructure and the high cost equipment in the private sector; and (c) long waiting times in the public sector for many services and examinations. The implementation of this law has been continuously postponed, mainly due to cost concerns but also due to lack of solid political will.^33^ The new schedule for its implementation has been delayed to the end of 2020, following a step by step approach.^24^

It is noteworthy that Cyprus has one of the lowest spends in terms of the percentage of gross domestic product (GDP) for healthcare in relation to other European countries.^16^ According to Ministry of Health data, total healthcare expenditures in Cyprus in 2016 accounted for 6.8% of GDP, divided between state budget (2.9% of GDP or 43% of total expenditure) and private sources (3.9% of GDP or 57% of total expenditure). It can therefore be characterised as a highly privatised system, as more than 47% of the total expenditure comes from out-pocket-payments, which is more than twice the European Union (EU) mean (20.2% in 2013), placing the country in the top position in Europe for private expenditure on healthcare.^16^ These payments mainly represent direct payments to the private sector, since the level of cost sharing arrangements remains at very low levels.^34^ In addition, it is estimated that only 0.06% of the GDP goes towards oral healthcare.^19^

Oral healthcare in Cyprus is currently provided on the one hand by the public dental services (PDS), and by the private sector on the other. Although more than 80% of the population could benefit from oral healthcare provided by the public sector, only a very low percentage is using these services.^8^ According to the Cyprus Statistical Service data^30^ in 2011 (2 years before the onset of the economic crisis), only 10–12% of the population were visiting PDS, while Eurobarometer survey findings on oral health (2010) indicated that 91% of Cypriots stated that they favoured private rather than public dentists.^14^ That was because the range of public sector services does not include costly treatment such as fixed prosthetics and implants, and also because people believe that the infrastructure, technology, materials used and overall quality of private sector services are better.

Public dental care is financed from the budget of the Ministry of Health. As a result of the previously mentioned austerity measures, the Ministry’s budget has shrunk and consequently the PDS funding has been reduced by 21.8% during the last 5 years. This reduction is mainly due to salary cuts which correspond to about 80% of the PDS budget.^26^

In contrast to the small numbers of dentists who work in the PDS (n = 40, 4.7%), more than 800 dentists work in the private sector, usually in solo practices and, as previously stated, they are remunerated directly by patients from out-of-pocket payments. The 1:1005 ratio of dentists per inhabitant in Cyprus is one of the highest in the EU (the EU average was 1:1450 in 2015) suggesting a tendency for professional saturation.^19^ Although there is no Dental School in Cyprus at the moment, it is estimated that every year more than 20 new dentists are entering the oral healthcare market, mostly after studying in EU countries, mainly in Greece, UK and Hungary. The expected opening of a Dental School in Cyprus by a private university will further aggravate the situation, leading to an even greater oversupply of dentists.

This large number of dentists is distributed all over Cyprus, ensuring easy access to dental care, even in small rural and remote communities. This is confirmed by the findings of a Eurobarometer survey, according to which 96% of Cypriots stated that they could visit a dentist within 30 minutes of their home. This percentage was the highest among EU countries.^14^

In order to practice dentistry in Cyprus, graduates of dental schools must obtain licence from the Cyprus Dental Council, the seven members of which are appointed by the Council of Ministers. Cyprus Dental Council is the competent authority for the registration of dentists in Cyprus as well as for the recognition of dental specialities. Additionally, all dentists must be registered with the Cyprus Dental Association.

Unlike in Greece, all public sector dentists, as well as the vast majority of private sector dentists, work with a dental assistant (dental nurse). Dental assistants in Cyprus do not have any special training, although some hold a diploma as a dental technician. The licence to practise the profession of dental technician is obtained from the Dental Technician’s Council, and requires 4-year training in a recognised educational institution. In 2015 there were 280 registered dental technicians.

The recognised specialties in Cyprus are those of orthodontics, oral and maxillofacial surgery and oral surgery. More specifically, in 2016 there were 50 registered orthodontists, 20 oral and maxillofacial surgeons and 7 oral surgeons. Recently, efforts have been made for the recognition of the specialities of endodontics, periodontics and paediatric dentistry.^22^

Thus, the aim of this paper is to present recent developments regarding the provision of oral healthcare in Cyprus and also to describe the impact of the economic crisis on the oral health of the Cyprus population.

## MATERIALS AND METHODS

Data from oral health surveys in Cyprus over the last 30 years were reviewed and analysed together with policy documents. In 2014 a survey of random samples of the population and dentists was performed using two questionnaires (one for the general population and one for dentists). The sample sizes (500 adults aged 35–44 years old and 500 adults aged 65–74, corresponding to 0.5% and 0.7%, respectively, of the referred populations, as well as 300 dentists, corresponding to 35% of the registered dentists in Cyprus) were calculated using a power calculation. The questionnaires, which included closed questions, were piloted and revised before they were distributed to the potential respondents, together with a letter explaining the nature of the survey and confirming that responses would be confidential and none of the responders would be identified when the results of the survey were published.

All the necessary validity and reliability checks of the questionnaires were conducted including content and face validity as we all as test-retest reliability (content validity score was 1 while Pearson’s correlation coefficient and Spearman’s rank correlation coefficient ranged between 0.7 and 0.8 indicating medium to strong correlation). The questionnaires were sent by e-mail to the dentists while those for the population were completed when they visited a dentist for an oral examination. The questions that were asked appear in the tables for this paper and were on the topics of oral health behaviour, dental visits and the consequences of the economic crisis on the latter. Approval for the survey was given by the Ministry of Health and the Cyprus Ethical Committee. The response rates were 91.2% (n = 456) for adults aged 35–44 years old, 95% (n = 475) for people aged 65–74 and 51% (n = 153) for dentists.

The results of the study and the reviews of the results of oral health surveys and policies are reflected in the sections of this paper.

## RESULTS

### Economic Crisis, Demand and Provision of Dental Care

According to a recent survey^27^ it was found that the economic crisis has negatively affected citizens’ behaviour in seeking dental care and visiting dentists in Cyprus. This change in behaviour is reflected in their responses to seven questions on seven different aspects of dental care (Table 1). The most important finding was that more than one in three participants stated that ‘during the last 12 months had visited a dentist less often in relation to the past’. This behavioural change occurred to a greater extent in the elderly as well as in people working in lower level of occupations (OR = 4.53, p <0.001), leading to increasing inequalities in oral health. In addition, the same study identified a shift of patients from the private to the public sector, especially among older people as 7.4% of people aged 35–44 and 17.3% of people aged 65–74 stated that ‘during the last 12 months had changed their private dentist opting for a public sector one’.^27^

**Table 1:** Positive responses by citizens regarding the impact of the economic crisis in seeking oral healthcare in Cyprus by age group (2013)

Question	Age group n (%)	
	35–44	65–74
During the last 12 months have you visited a dentist less often compared with the past?	122 (33.2)	109 (37.1)
During the last 12 months have you visited a dentist only for an emergency compared with the past?	98 (26.7)	97 (33.0)
During the last 12 months have you asked your dentist for a discount compared with the past?	52 (14.2)	112 (38.3)
During the last 12 months have you asked for a more economic plan?	50 (13.6)	74 (25.2)
During the last 12 months have you changed your private dentist opting for a public sector one?	27 (7.4)	51 (17.3)
During the last 12 months have you paid your dentist in instalments compared with the past?	27 (7.4)	38 (12.9)
During the last 12 months have you cancelled a scheduled appointment due to economic reasons compared with the past?	25 (6.8)	24 (8.1)

The findings regarding citizens’ oral healthcare largely coincided with those coming from the responses of dentists to the same survey, confirming the change in their behaviour in seeking dental care and meeting their needs. For example, it was found that 37.2% (n = 48) of the private sector dentists stated that during 2013 the number of new patients had ‘decreased or much decreased’, while the percentage of those who stated that visits were reduced was even higher, approaching nearly half of the dentists (47.7% n = 61) and increased numbers of patients asking for payment by instalments (Table 2).^9^ Moreover, even higher reduction rates were recorded for high-cost dental work such as implants, and fixed prosthetics. In 2013, 73.9% (n = 82) of private dentists reported a decrease in demand for fixed prosthetics compared with the previous years. This percentage fell to 53.4% (n = 55) in 2015 (p = 0.6). The corresponding percentages for the demand of implants were 73.3% (n = 50) and 65.4% (n = 27).

**Table 2:** Positive responses of private sector dentists regarding the patients’ oral health behaviour the years 2013 and 2015

Question: In relation to the previous years the …	Increased or much increased n (%)		Remained stable n (%)		Decreased or much decreased n (%)		p (a)
	2013	2015	2013	2015	2013	2015	
Number of new patients	31 (24.1)	54 (43.9)	50 (38.8)	29 (23.6)	48 (37.2)	40 (32.6)	0.005
Number of patients who cancelled appointments	73 (57.4)	37 (30.1)	44 (34.6)	69 (56.1)	10 (7.8)	17 (13.8)	0.9
Number of patients who did not pay part or the whole amount of the treatment	75 (58.2)	72 (48.5)	47 (36.7)	39 (31.7)	6 (4.7)	12 (9.7)	0.6
Number of patients who sought a discount	122 (94.6)	109 (88.6)	7 (5.4)	10 (8.1)	0.0	4 (3.3)	0.8
Number of patients who asked for payment by instalments	125 (96.9)	109 (87.0)	4 (3.1)	15 (12.2)	0.0	1 (0.8)	0.9
Number of patients who postponed a treatment due to economic problems	117 (90.7)	97 (78.8)	12 (9.3)	22 (17.9)	0.0	4 (3.3)	0.7
Daily number of patients	15 (11.7)	37 (30.1)	52 (40.6)	47 (38.2)	61 (47.7)	39 (31.8)	0.07

a = x2 test.

The situation appears to have improved in 2015, when 43.9% (n = 54) of dentists who took part in the survey reported that the number of their new patients increased compared to 24.1% (n = 31) in 2013 (p = 0.005) and 30.1% (n = 37) that the daily number of patients seen increased compared to 2013 (Table 2).^9^

The reduction of in the numbers of patients and visits, and the fall in demand for costly dental work led to a fall in the private sector dentists’ income. Thus, 81.3% (n = 104) of the private dentists who were surveyed, stated that their income decreased in 2013 compared to previous years. As the economic crisis is diminishing, and 2 months before the country’s exit from the memorandum (January 2016), the private dentists who were surveyed appeared more positive, as 25.2% (n = 31) stated that their income increased in 2015 compared to previous years (the corresponding percentage for 2013 was just 4.7%), while to the contrary, only 49.7% (n = 61) stated that it had decreased (compared to 81.3% (n = 104) in 2013). However, the difference over the 2-year period of the impact of the economic crisis on private sector dentists’ income is not statistically significant (p = 0.08), and reflects a gradually improved economic environment. Besides, almost 50% of private dentists continued to report a further decrease of their income.^9^ In contrast, dentists working in the public sector, both in 2013 and 2015, stated that they felt much stress and pressure due to the increased workload and the many patients they have to treat. This fact seems to have a negative impact on the quality of services they offered, which they assessed less positively in relation to the private sector dentists’ assessment.^9^ More specifically, in 2013 one in two private sector dentists who were surveyed assessed the quality of their work as better in relation to previous years, in comparison to only 26% of public sector dentists (x^2^ = 4.3, p = 0.038). Another cause of stress for public sector dentists has been the reduction in their salaries, which was almost 30% in 2013 compared to the past. At the same time, the expressed professional insecurity is more than obvious (20.8% in 2013, a percentage that increased to 28.6% in 2015), probably not only due to the reduction of their income, but also the role of the PDS within the new National Health System which has not been clarified.^9^

### Epidemiological Studies

Epidemiological studies over the last three decades in Cyprus have shown a reduction in caries prevalence and the dmft/DMFT index for children of 6, 12 and 15 years.^4,6,7^ The most recent study, which was conducted in 2014 showed that the dmft/DMFT for the three age groups was at relatively low levels as shown in the last column of Table 3.^10,27^

**Table 3:** Trends in the caries indicator in Cyprus for different age groups (1992–2014)

Age group	DMFT			
	1992	2005/06	2010	2014
6 (dmft)	–	2.21	2.14	1.83
12	2.16	1.14	1.25	1.26
15	3.87	–	1.98	1.98
35–44	13.32	–	–	10.24
65–74	17.76 (people 55–64)			16.93

Despite the relatively low values of mean dmft/DMFT there were wide variations among different districts. Higher levels of caries for all age groups were recorded in the Larnaca district and lower in the Paphos district. The corresponding dmft/DMFT values for the age groups of 6, 12, 15, 35–44, 65–74 for Larnaca district were 2.93, 1.92, 2.48, 13.29, 19.37, while for Paphos these were 0.93, 0.76, 1.41, 9.65, 16.92, respectively. Statistically significant differences were observed among different socioeconomic groups. In particular, it was found that children whose parents worked in low-level occupations had higher dmft/DMFT scores (OR = 1.38 p = 0.049) and more treatment needs (OR = 0.35, p = 0.001). On the other hand, it was found that children with parents who worked in high-level occupations visited a dentist more regularly and mainly for preventive reasons (OR = 2.20, p <0.001).^27^ Variations were also observed among different ethnicities, with children of immigrants from Eastern Europe recording a higher prevalence of dental caries (OR = 1.89, p <0.01).^10,27^

Only 17.4% (n = 97) of the 12-year-old and 15.3% (n = 85) of the 15-year-old children had sealants. In addition, orthodontic appliances were found in 9% (n = 50) of the 12-year old and 20.9% (n = 106) of the 15-year-old children. The lower occupational level of parents was positively related to less frequent existence of sealants in the mouth (OR = 3.30, p <0.001) and to the non-existence of orthodontic appliances (OR = 2.47, p <0.001)

Regarding the community periodontal index of treatment needs (CPITN) index, only 46.1% of the 12-and 34.8% of the 15-year-old children had healthy gums (CPITN = 0) despite the fact that 53.5% of the 12-year-old and 43.8% of the 15-year-old children claimed that they brushed their teeth twice a day.

Regarding school absenteeism, 9.2% (n = 51) of the 12-year-old and 9.1% (n = 46) of the 15-year-old children mentioned that they had to be absent from school due to dental problems, with an average time loss of 5.63 and 6.74 h/year, respectively. A similar percentage of people aged 35–44 years old (8.1%, n = 37) reported absence from their work due to oral health problems. However, the average loss of time was higher at 9.8 h per year, mainly due to the more severe problems the adults are facing compared to the children.

The level of adults’ oral health can also be considered as satisfactory. More specifically, the factor missing teeth for adults aged 35–44 years old accounted for 27.2% of the overall DMFT index in 2014 compared to 43.1% in 1992. However, 41.4% of the people aged 35–44 years and 46.5% of the people aged 65–74 years recorded unmet dental needs (DT>0) and, respectively, 20.6% and 41% with unmet need for prosthetic rehabilitation.

As with children, adults with low level occupations or a low level of education recorded higher levels of dental caries, more treatment needs and visited a dentist less often.

In relation to the community periodontal index (CPI) and its components parts, although improvement has been found over time, deterioration is also apparent as the population grows older, since 28% of those aged 65–74 years were found to have shallow pockets (3–5 ml) and 7% deep pockets (>5 ml) (Table 4). These data are consistent with international literature according to which the severe form of periodontitis affects 10.8% of adults and constitutes the sixth most chronic condition.^17^

**Table 4:** CPI indicators during 1992 and 2014 for different age group

Age group		CPI (%)				
		0	1	2	3	4
12-year-olds	1992	23.8	46.9	28.7	0.0	0.0
	2014	46.1	38.4	14.2	0.0	0.0
15-year-olds	1992	39.1	33.6	26.7	0.0	0.0
	2014	34.8	35.9	28.9	0.0	0.0
35–44	1992	9.2	9.4	60.9	19.1	1.5
	2014	22.2	23.3	42.1	11.7	0.7
65–74	1992 (55–64)	2.5	6.8	43.9	42.4	4.5
	2014	8.4	16.3	40.3	28.0	7.0

## DISCUSSION

As in other Mediterranean European countries, in Cyprus oral healthcare is mainly provided by the private sector and is paid from the household budget. The recent economic crisis has negatively affected the demand and provision of oral healthcare. Similar phenomena were also observed in other EU countries that have been affected by the economic crisis, such as Greece,^12^ Ireland,^31^ Italy,^2^ and Spain^3^ as well as in the USA.^20,21,23^

More specifically, the economic crisis has led to the following consequences in oral healthcare provision in Cyprus:

a) Reduction in the number of dental visits and treatment provided by the private sector, in an effort to reduce private spending leading to a sharp decline in the income of private dentists over the last years. In a similar study conducted in Greece in 2009,^18^ in the midst of the economic crisis, a statistically significant percentage of individuals stated that they restricted their visits to doctors and dentists. Confirmation of the negative impact of the economic crisis also comes from the findings of a study in Ireland, according to which 23% of interviewed dentists stated that as a result of the economic crisis, they had fewer visits to their practice in 2013 in comparison with 2010,^31^ while in Italy, 90% of the Presidents of the local associations stated that as a result of the economic crisis in 2013 they had fewer patients in comparison with 2008 and 95.3% of them reported an income reduction in 2013, compared with 2008 due to the economic crisis.^29^

b) A shift of patients from the private to the public sector. However, the recorded shift of patients from the private sector to the PDS, after the onset of the economic crisis, seems at first glance to be in conflict with the statistical data regarding the utilisation of PDS which showed that the continuous increase of visits in previous years, due to improvements of infrastructure and equipment, was interrupted in 2013 after the introduction of copayments.^26^ This observation can be explained because of two possible causes: (a) the new patients coming from the private sector, possibly turned to the PDS temporarily, making very few visits, and only for emergencies; and (b) the economic crisis and the increased copayments had a greater impact on reducing visits in relation to the increase of visits as a result of entitled patients shifting to the public system. An indirect confirmation of this claim could be based on the higher percentage of Cypriots who reported unmet dental needs in 2014 in comparison with 2013 (10.4% and 8.8%, respectively), while in the same period the EU-28 recorded a slight decrease (7.8% vs 7.6%).^15^

However, in spite of the economic crisis, recent epidemiological studies show that the level of oral health is still at satisfactory levels, and is comparable to that of other developed European countries which spend more resources for oral health.^5^ Twenty-four years after the last study in 1992, not only was a steady improvement in the level of oral health recorded but also an improvement of all components of the DMFT index and especially that of the missing teeth. This may be attributed to the overall lifestyle changes in that lead to the adoption of better oral hygiene and nutrition habits, as well as to the effective use of fluorides. Yet, oral health disparities still exist, a phenomenon similar with other developed countries with children and adults from lower socioeconomic strata, as well as children from an immigrant background, recording higher caries index, more treatment needs and visiting a dentist less often.^2,3,12^ The impact of various socioeconomic factors on the caries index and on treatment needs was further confirmed through the Eurostat data where in 2014, 10% of Cypriot citizens who found themselves in the first (lowest) quartile regarding their income reported unmet dental needs due to the cost of dental treatment in comparison with 1.5% of individuals in the fifth (highest) quartile (mean average 5.1%).^15^

Certainly, it’s too soon to draw any final conclusions on the medium- and long-term consequences of the recent economic crisis on the level of oral health of Cypriots, especially having in mind that the new National Health System (which is expected to be implemented by 2020) will provide only preventive dental services for children up to the age of 16 years, such as examination and topical application of fluoride. Although the promotion of preventive services for the child population can be considered as a positive measure, bearing in mind that the percentage of children with sealants is quite low in Cyprus compared to other countries (such as Denmark, where over 10 years ago, 68% of the 15 year old children had at least one fissure sealant in their mouth,^13^ or in the USA, where in the years 1991–2002, 32% of children aged 6–19 had fissure sealants),^1^ it is obviously inadequate, especially for the low socioeconomic strata of the population who can’t afford go to the private sector and who are currently using the PDS, and is expected to further widen the existing inequalities in oral health. The Irish experience of limiting access to dental care due to the economic crisis, led to deterioration in the population’s oral health level.^31^

Considering these data and bearing in mind the importance of oral health and its interaction with general health and quality of life, it is necessary to rethink and redesign the strategy for oral health. Despite the current economic difficulties, Cyprus should consider and provide all oral healthcare services to young people up to 18 years, in the context of the new health system, which is a common policy for most European countries. In addition, it must ensure access to basic dental care services at least for the lower socioeconomic strata. Thus, the size, structure, organisation and therefore the role of the PDS under the new NHS should be determined on the basis of the above. Within this framework, upgrading of infrastructure and dental technology, better staffing and quality of services will convince beneficiaries that the services provided are as good as those of the private sector.

For a further and continuous improvement of oral health, the participation of dentists is not enough. Family doctors and all other medical specialties and health professionals working in primary care must be involved. In addition, a multidisciplinary approach is also needed for the promotion of oral care, due to the strong correlation of different socioeconomic factors, oral diseases and treatment needs.^25,28,32^ Therefore, any effort for the promotion oral health has to include broader socioeconomic measures so that the deeper causes of oral diseases can be dealt with.^35^

## CONCLUSIONS

Recent epidemiological data have shown that the oral health of people living in Cyprus has improved to a level comparable with that of other developed EU countries. However, the impact of the 3-year economic crisis has negatively affected the behaviour of citizens in seeking oral healthcare and meeting their needs. Although this change may have a negative impact on oral health, it is impossible to estimate accurately any changes in both indices and inequalities. The data presented in this paper highlight the new challenges that both the public and private sector in Cyprus face in relation to the provision of oral health services into a new and competitive environment, which in the coming years will be created by the introduction of a new national healthcare system.
